# Enhanced electrochromic switching contrast in the blue by 3,4-propylenedioxypyrrole – implementation on structural colors

**DOI:** 10.1515/nanoph-2022-0624

**Published:** 2023-01-12

**Authors:** Oliver Olsson, Marika Gugole, Andreas Dahlin

**Affiliations:** Department of Chemistry and Chemical Engineering, Chalmers University of Technology, 41296 Gothenburg, Sweden

**Keywords:** conductive polymers, reflective displays, structural colors

## Abstract

Recent advances in nanofabrication technologies have enabled new ways to produce structural colors. By combining nanofabrication methods, it is possible to integrate electrochromic materials with the nanostructures, which enable electrical tuning of the colors and thus new types of reflective displays. Previous work has shown high quality colors and high switching contrast in general. However, so far the intensity modulation has always been more limited in the blue. In this work we prepare blue structural colors and synthesize films of an electrochromic polymer (PProDOP) that is optimized for high contrast in this spectral region. A protocol for electropolymerization of PProDOP on gold surfaces is presented. The polymer films are shown to follow Lambert–Beer behavior and can provide up to 75% contrast (difference in transmittivity). On blue nanostructures, the reflectivity can be modulated with a contrast of 50%, which is a considerable improvement in comparison with previous work. The results presented here should be useful for electrochromic or other electro-optical devices operating in the blue spectral region.

## 1 Introduction

The combination of structural colors and electrochromic materials is currently attracting a lot of attention [1–8]. One of the most promising applications is displays which reflect light from the environment, thereby consuming minute amounts of energy compared to their emissive counterparts (mainly LCD and LED displays). Similar electrochromic devices are also of interest for several other applications [3, 8]. Both organic and inorganic electrochromic materials have been explored for tunable structural colors. While the former are based on polymers with *π*-conjugation in the backbone, the latter consists of transition metal oxides. In practice, both materials are implemented in the same manner, where the surface with the electrochromic nanostructure makes up one of the electrodes in an electrochemical cell [4].

**Figure 1:**
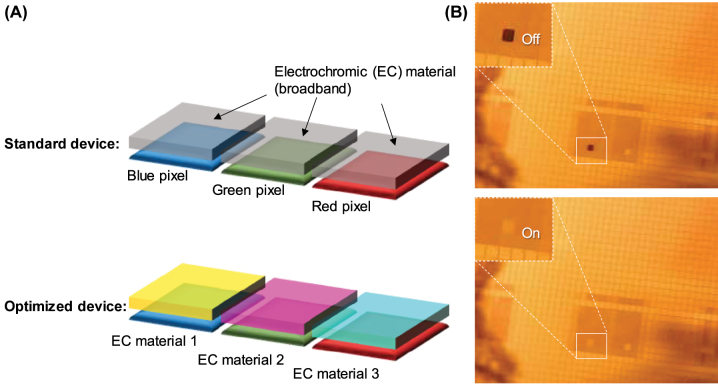
Motivation for the work. (A) In a reflective display where color mixing is achieved by subpixels (here RGB), performance can be increased by using different electrochromic materials for the different primary colors, since no single material is “perfect broadband”. In particular, there is a need to find good materials for switching blue pixels with high contrast. (B) Proof-of-concept for electropolymerization and addressing of individual gold pixels (200 µm × 200 µm) in an active matrix. A single pixel where PProDOTMe_2_ has been synthesized [9] is switched.

One of the most central benchmark parameters in electro-optical devices is the switching contrast, i.e. the magnitude of the intensity modulation, normally defined as the difference in transmittivity (*T*) or reflectivity (*R*). In general, high-contrast switching (∆*T* or ∆*R* > 50%) has been achieved using both organic and inorganic materials [9]. However, in the blue spectral region (400–500 nm) the contrast has been lower than in the rest of the visible. This is mainly because the same electrochromic material has been used to modulate all colors and there are significant spectral variations in the magnitude of the intensity changes. Importantly, it is possible to prepare different electrochromic materials on different structurally colored pixels in a display device. Using electropolymerization, there is not even a need for lithography steps to achieve this goal: if individual pixels or segments can be electronically addressed, one can synthesize conductive polymers on them selectively [10]. Thus, instead of using a single polymer (or metal oxide) for all pixels/colors and aim for high-contrast broadband switching, there is a clear value in finding different polymers that provide maximum contrast only in a spectral region corresponding to a single color. For instance, a device with red, green and blue (RGB) pixels could have three different polymers, each giving maximum contrast for one primary color (Figure 1). While polymers such as polypyrrole [10] (PPy), poly(3,4-ethylenedioxythiophene) [11] (PEDOT) and poly(dimethylpropylenedioxythiophene) [9] (PProDOTMe_2_) work well for electrochromism in the green and red regions, poly(3,4-alkylenedioxypyrroles) are potentially interesting to use in the blue [12]. However, to date this class of conductive polymers have not been implemented with structural colors, or for active tuning of other resonant nanostructures.

In this work we present a recipe for electropolymerization of poly(3,4-propylenedioxypyrrole) (ProDOP) on gold. The optical properties of the nanoscale films are characterized and important factors influencing the switching contrast are identified. We show that the electropolymerization can be performed directly on blue-colored nanostructures fabricated over large areas, enabling improved contrast for switching in reflection mode. This work is important for improving the performance of electrochromic and other electro-optical devices operating in the blue region.

## 2 Experimental

Chemicals: Propylene carbonate (PC, ReagentPlus^®^ 99% and anhydrous 99.7%) and lithium perchlorate (LiClO_4_, battery grade, dry, 99.99% trace metals basis) were purchased from Sigma-Aldrich. The lithium perchlorate and the anhydrous propylene carbonate were stored in a glove box. Tetrabutylammonium perchlorate (TBAP, for electrochemical analysis, ≥99.0%) was purchased from Supelco. Propylenedioxypyrrole (ProDOP >95%) was purchased from Accela ChemBio. No further purifications were made for any of the chemicals. The electrolyte for electrochemical switching was prepared in a glovebox and degassed using N_2_.

Fabrication of metasurfaces: Al (100 nm with 5 nm Ti adhesion layer), Al_2_O_3_ (95 nm), and Au (20 nm with 1 nm Cr adhesion layer) were deposited by evaporation (Lesker PVD 225) onto glass substrates. Prior to Au deposition, colloidal lithography was performed on Al_2_O_3_, using 147 nm polystyrene-sulfate particles (Microparticles GmbH). Samples for transmission measurements (20 nm Au with 1 nm Cr adhesion layer) were prepared by evaporation onto glass.

Electropolymerization: All experiments were carried out in a custom-made spectro-electrochemical cell (Redox Me AB) with a Pt coil as a counter electrode and an Ag electrode in contact with 0.01 M AgNO_3_ and 0.1 M TBAP as reference (Ag/Ag^+^). The flow cell was cleaned with ethanol and dried with N_2_. Prior to use, the substrate was rinsed with acetone and isopropanol then dried with N_2_. The polymerization was carried out in a solution consisting of 10 mM monomer and 0.1 M TBAP-PC (ReagentPlus 99%). The solution was degassed with N_2_ purge for 10 min. Water (MilliQ, degassed with vacuum and sonication) was added to a concentration of ∼1500 ppm, as determined by Karl-Fischer titration (Mettler Toledo C20S). A constant potential of +0.6 V versus Ag/Ag^+^ was used for all polymerizations. For characterization, FTIR spectra of the polymer films were measured in ATR mode with a Perkin Elmer Frontier. The polymer film on the substrate was cleaned by dipping the sample in isopropanol and immediately shaking it vigorously in the solvent.

Electrochemical measurements: The polymer films were switched between −1.2 V and 0 V versus Ag/Ag^+^ in 0.1 M LiClO_4_ in propylene carbonate (anhydrous 99.7%) using a potentiostat (Gamry Reference 600+).

Optical measurements: A home-built microscopy/reflection/transmission setup [9] was used with a tungsten lamp (Azpect Photonics) illuminating the sample. The transmitted/reflected light was collected with an objective (Olympus, 4× air NA 0.1) and collected by a spectrometer (B&WTek). The dark counts in the detector were always subtracted from measured intensities. For transmission measurements, the reference intensity was that through a gold film without any polymer. For reflection measurements, the reference was a mirror (BB05-E02, Thorlabs). To obtain the reflectivity of the nanostructure only, the spectra were corrected with respect to the reflection from the flow cell window and electrolyte [9]. (The reflectivity of a silver coated surface was measured inside and outside the flow cell). Images of illuminated spots were captured with a Thorlabs DCC1645C CMOS camera.

## 3 Results and discussion

**Figure 2:**
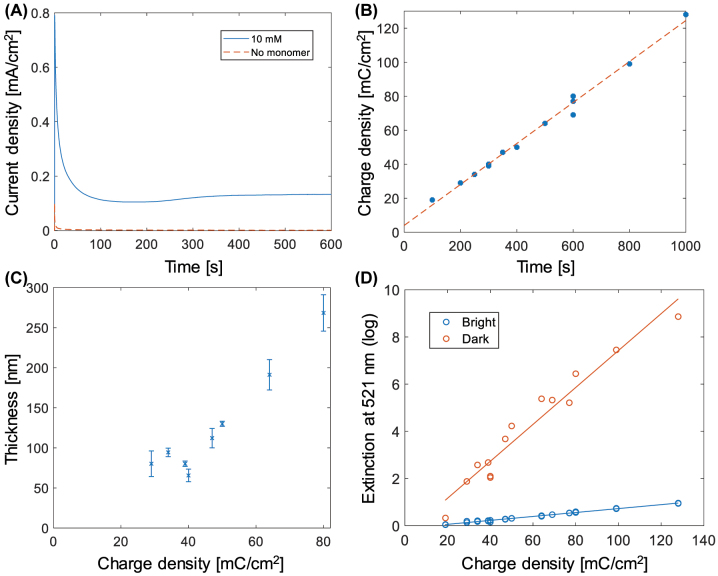
Preparation of PProDOP films by electropolymerization. (A) Chronoamperometry during electropolymerization of 10 mM ProDOP in 0.1 M TBAP in PC. A potential of +0.6 V was applied at time zero. (B) Integrated current as a function of polymerization time (each data point is one sample). The line shows a linear fit. (C) Thicknesses measured by profilometer for different polymerization times represented as charge transferred. The variation is high for the thinnest films but a linear trend emerges for thicker layers, suggesting ∼3 nm thickness per mC/cm^2^. (D) Extinction at 521 nm (wavelength of maximum absorption) in dark and bright states for films of different thickness (represented as charge density from polymerization).

The preparation of nanoscale electrochromic PProDOP films is presented in Figure 2. Our focus lies on the electropolymerization process and its implementation on conductive nanostructures rather than the synthesis of the monomer [13, 14] (which was purchased from a supplier). Note that electropolymerization also works for the case of separate metal nanoparticles on a support since transparent conductors such as indium tin oxide (ITO) can be used to make the structure conductive [15]. For details about the mechanism of PProDOP electrochromism we refer to previous literature [12]. In this work, one of our aims was to obtain high quality polymer films on gold surfaces, since this metal is commonly used for structural colors.

Importantly, while electropolymerization was straightforward on indium tin oxide (ITO) similarly to literature protocols [16], we found that on gold the recipe needed careful optimization to obtain high quality films. Electropolymerization was performed by applying a constant oxidative potential in a 0.1 M tertbutylammonium perchlorate (TBAP) electrolyte in propylene carbonate (PC). This eventually led to a steady current density of 0.12 ± 0.015 mA/cm^2^ in the presence of 10 mM of the monomer (Figure 2A). The steady current lead to a linear relation between polymerization time and charge transfer (Figure 2B), strongly suggesting a linear growth rate of the polymer film thickness. Profilometer measurements (Figure 2C) confirmed this and suggested a “thickness per charge” relation of ∼3 nm per mC/cm^2^. However, precise thickness measurements are difficult for these nanoscale thin films and throughout the rest of this paper we present the charge transferred during polymerization as an effective measure of polymer surface coverage.

We emphasize that the type of PC solvent and the water concentration during polymerization were important for the film quality. Interestingly, while it is practically impossible to fully avoid spontaneous uptake of water by PC, we found that the best films were *not* obtained with as little water as possible, but rather at concentrations of ∼0.15% (as measured by Karl Fisher titration). This is likely related to the fact that water is known to influence the polymerization of pyrroles in acetonitrile [17, 18]. Note also that voltage sweeps should not be used for the synthesis. The Supporting Information contains additional results and discussion on the electropolymerization.

As expected [16], the polymer was transparent after polymerization with ClO_4_^−^ ions, while it became yellow when a reductive potential was applied in a LiClO_4_ electrolyte due to absorption in the blue. Following the approach we focus on here (Figure 1) only the blue spectral region is of interest for modulation of blue light and the rest of the spectrum can essentially be ignored. In other words, it does not matter if the absorbing state is yellow rather than black, as long as it provides high contrast. We found that the extinction of the nanoscale polymer films was linearly dependent on polymerization time for both oxidation states (Figure 2D), as expected for Lambert-Beer absorption behavior. The experimental variation of the absorption is slightly higher than what we previously observed for other polymers [9] mainly because different surfaces had to be analyzed for each data point. Restarting the polymerization was not straightforward because unadhered polymer diffused into the electrolyte and altered the background intensity. Also, another electrolyte was used for the switching tests (Supporting Information). For these reasons it was more practical to rinse and dry the sample after polymerization, then remount it for switching tests.

The Lambert–Beer behavior implies that the highest contrast that can be achieved is determined solely by the ratio of the extinction spectra *E*(*λ*) in both oxidation states according to [9]:

(1) $$\Delta T_{\text{max}}={\left[\frac{E_{\text{re}}}{E_{\text{ox}}}\right]}^{\frac{1}{1-E_{\text{re}}/E_{\text{ox}}}}-{\left[\frac{E_{\text{re}}}{E_{\text{ox}}}\right]}^{\frac{E_{\text{re}}/E_{\text{ox}}}{1-E_{\text{re}}/E_{\text{ox}}}}$$

We define *E* as equal to log(*I*_0_/*I*) with natural logarithms, where *I* is the measured intensity and *I*_0_ is the reference intensity (no polymer on the gold). Note that the extinction is logarithmic, while the transmission is simply *T* = *I*/*I*_0_. Using the relation between thickness and charge transfer from (Figure 2C), the absorption capacities *α* at 521 nm were determined to be 0.026 nm^−1^ and 0.0028 nm^−1^ in the colored and bleached states respectively. (*E* = *αd* where *d* is the film thickness).

**Figure 3:**
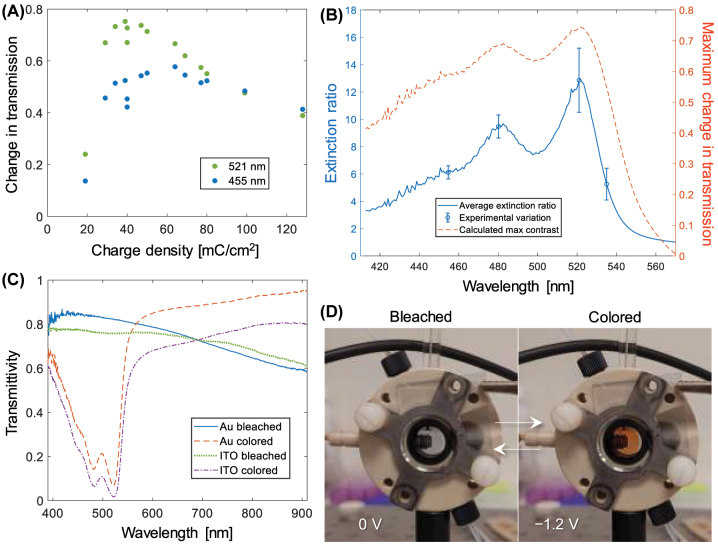
Contrast optimization. (A) Change in transmission between oxidized and reduced states for PProDOP films of different thickness (represented as charge transfer during polymerization). Data shown for the wavelength of maximal contrast (521 nm) and for blue (455 nm). All samples were prepared on gold. (B) Spectrum of the average extinction ratio. The error bars (two standard deviations) show the typical variation when including multiple samples with different film thickness. The right axis shows the maximum contrast predicted from the extinction ratio according to Equation (1). (C) Example transmission spectra for PProDOP in both oxidation states on gold and ITO. (D) Photos of PProDOP switching in an electrochemical cell.

Figure 3A shows the difference in transmission at the different oxidation states measured at 521 nm for different polymerization times. A clear optimum (for 521 nm wavelength) can be identified for polymerization times around 300 s, which corresponds to a charge transfer of ∼40 mC/cm^2^ and a film thickness of ∼100 nm. Figure 3B shows the corresponding spectrum of the extinction ratio, which should remain constant if *E* is proportional to film thickness. Yet the extinction ratio did show some variation (see error bars in the plot). This stems from a variation of the electrochromic performance of each sample, which could be due to variations of the exact water content during electropolymerization or the thickness of the supporting gold film, as each data point corresponds to a separate surface and polymerization run. Alternatively, the polymer might exhibit complex swelling/contraction behavior which is dependent on the thickness. Strictly, Equation (1) is only valid if there is no swelling or if the change in thickness of swollen/compact states is described by a constant ratio [9]. Regardless, we consider the average extinction ratio from multiple samples as a good measure of the contrast that can be achieved with PProDOP. (Individual samples may perform even better). Figure 3B also shows a spectrum of the highest contrast that can be achieved based on Equation (1) and the measured extinction ratio spectrum. The typical maximum contrast is about 75% at a wavelength of 521 nm. This corresponds to green rather than blue, but the whole blue region (400–500 nm) also exhibits a high contrast, suggesting that the polymer is suitable for blue electrochromism. For comparison, other yellow-transparent polymers have reached 70% contrast in transmission mode [19]. Note that the thickness which gives highest contrast is wavelength dependent. Assuming that 455 nm is a good wavelength to represent “deep blue” color, the data in Figure 3A indicates that a considerably higher thickness should be used for modulating blue light with optimal contrast (in comparison with the optimal thickness for modulation at 521 nm).

The transmission spectra of PProDOP in both oxidation states are shown in Figure 3C including films prepared on ITO for comparison. The spectra are clearly very similar, which suggests that our recipe provides high quality films also on gold, at least under optimal conditions (Supporting Information). Note that at wavelengths above 570 nm (for gold), the switching contrast is actually reversed, so that PProDOP is instead slightly more absorbing in its oxidized state. Yet to the eye the films appeared transparent rather than blue. Photos of PProDOP films in their different oxidation states on ITO are shown in Figure 3D.

**Figure 4:**
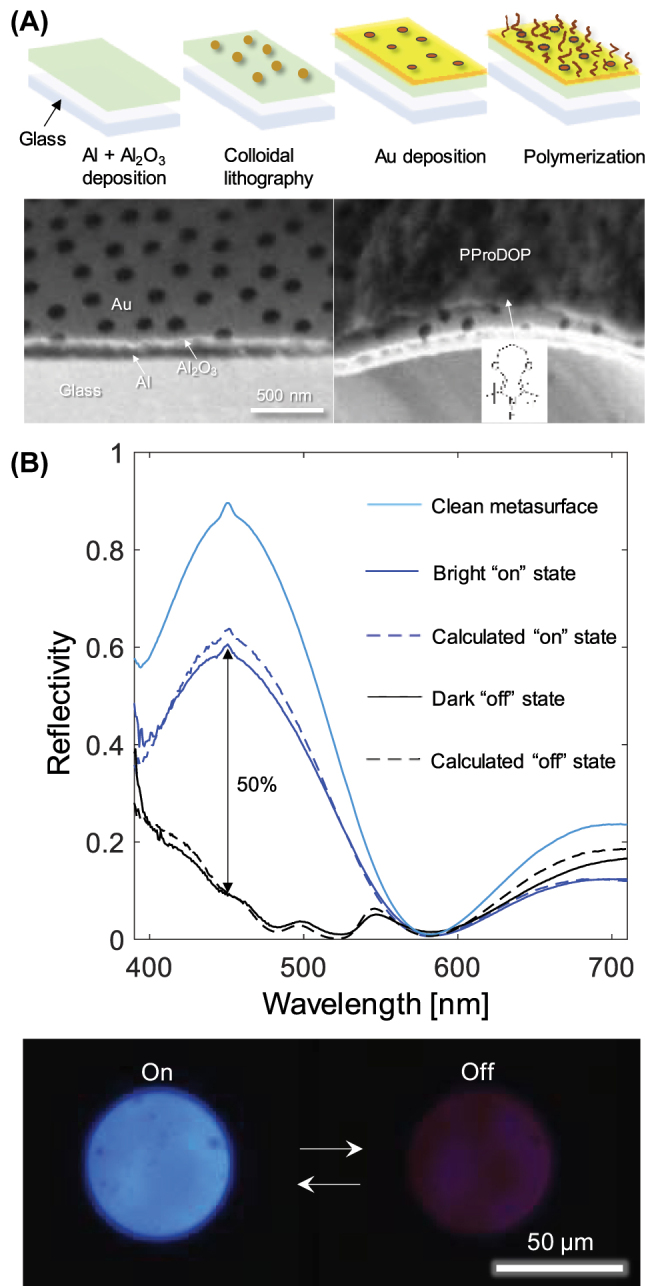
Implementation of PProDOP with structural colors. (A) Fabrication and electropolymerization to achieve electrochromic blue metasurfaces. The electron microscopy images show a cross-section of the nanostructure before and after electropolymerization. (B) Reflectivity spectra of the unmodified metasurface and after PProDOP electropolymerization for both oxidation states. The dashed spectra were generated by simulating a PProDOP film above (optically decoupled from) the metasurface. The maximum contrast is indicated. Images show the reflection from a blue metasurface with PProDOP in its different oxidation states. Note that the illumination intensity and camera settings are the same in both cases.

To implement PProDOP with plasmonic structural colors, we prepared metal-insulator-metal nanostructures with nanohole arrays as described previously [9–11] and then polymerized the PProDOP on the top gold film with nanoholes. The fabrication process is described in Figure 4A. The Al mirror, Al_2_O_3_ spacer and semitransparent Au film with nanoholes give clear blue colors by combining a Fabry–Pérot resonance with surface plasmons. While the cavity reflects mainly blue light, the nanoholes decrease the reflectivity in the red spectral region by resonant scattering at high angles, thereby ensuring a blue color rather than purple [9–11]. After electropolymerization, the polymer film on top of the nanostructure was clearly visible in electron microscopy (Figure 4A).

Figure 4B shows the reflectivity spectra of the nanostructure before and after functionalization with ProDOP and upon switching it between the two oxidation states. The maximum contrast is at least 50% at 455 nm and remains high throughout the blue region (400–500 nm). This result was obtained when the polymerization time was around 300 s, corresponding to thicknesses in the range 50–100 nm (Figure 2). Thicker (400 s polymerization) or thinner (200 s polymerization) films gave significantly poorer contrast (∼40% reflectivity change), which confirms the importance of controlling and characterizing the polymer growth for optimal contrast.

The intensity modulation we obtain here is considerably higher than what has previously been achieved with other polymers on blue nanostructures: PProDOTMe_2_ gives maximum 45% at 490 nm [9], PPy gives maximum 30% at 480 nm^10^ and PEDOT:PSS gives only 25% at 480 nm [11]. Note that these literature values of contrast are not only lower, but also measured at “less blue” wavelengths. Thus, our work shows a considerable improvement of switching contrast, even deep into the blue region. Also shown in Figure 4B is the simulated spectrum from a hypothetical “shutter” consisting of PProDOP above the nanostructure, i.e. a purely absorbing film that is optically decoupled from the surface, generated from the absorption data in Figure 3. The simulated spectrum is clearly in excellent agreement with the measured spectrum, which shows that effects from thin film interference inside the polymer film or coupling with the nanostructure are both negligible. This is not surprising since the plasmonic activity lies in the red spectral region, where the polymer does not alter its absorption much.

The color quality was characterized by calculating the CIE 2D coordinates, which were *x* = 0.163, *y* = 0.157 for the unmodified metasurface and *x* = 0.160, *y* = 0.156 for PProDOP in the bleached state, showing minimal effect from the polymer. (The dark state had *x* = 0.278, *y* = 0.182.) Note that although even higher color purity can be achieved with dielectric structures, this occurs at the expense of low brightness (absolute reflectivity) because less of the incident light reaches the viewer. Indeed, we have previously argued that plasmonic structural colors, such as the ones shown here, provide a good balance between brightness and chromaticity [20].

**Figure 5:**
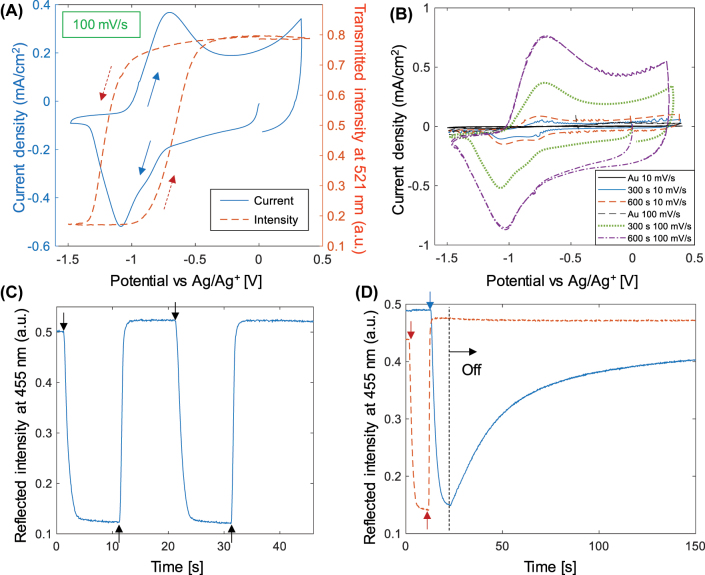
Additional characterization of PProDOP switching. (A) Cyclic voltammetry curve for a scan rate of 100 mV/s on 20 nm planar gold electrodes. The right axis shows the synchronized intensity trace at 521 nm during a voltage cycle. The arrows indicate the direction of changes during the sweep. (B) Additional CV data at two different scan rate and two different film thicknesses. (C) Switching dynamics of reflection at 455 nm from a blue metasurface. The arrows indicate when a steady voltage is applied (0 V or −1.2 V). (D) Maintained coloration state at open circuit (bistability). The bleached state is barely altered (dashed line) and is gradually recovered from the colored state over a timescale of ∼1 min (solid line).

The switching behavior of ProDOP was also characterized by voltammetry sweeps synchronized with the optical measurements. Figure 5A shows a typical cyclic voltammogram (CV) with the synchronized intensity at 521 nm. The optical response clearly saturates in both directions, i.e. it is not possible to increase the contrast further by going to lower or higher voltages. Also, the change in the optical signal coincides well with the redox activity detected by the current. Further CV scans are shown in Figure 5B for different film thickness. Note that it is sufficient to go to −1.2 V to fully reduce the polymer as long as it is given sufficient time to switch (which is the case at 10 mV/s). We did not observe any significant difference in the current measured from PProDOP on metasurfaces compared to planar gold.

Finally, we characterized the switching dynamics and lifetime of the electrochromic devices. Figure 5C shows the intensity time trace when altering the voltage with a square wave. The complete switch (reaching >90% of the intensity change) takes around 1 s, which we consider quite standard for organic electrochromism. This is for the PProDOP film thickness used on the blue nanostructures (thicker films are generally slower). Note that the switch speed can be increased in various ways, for instance by changing the electrode configuration, electrolyte etc. [21] (In this work we did not implement any such improvements.) Figure 5D shows the bistability of the system, i.e. the possibility to maintain a certain coloration state at open circuit. After switching to the yellow state and disconnecting, the polymer gradually returned to the transparent state over a timescale of ∼1 min. The bright state could be maintained essentially indefinitely, which suggests that this is the equilibrium state for the polymer. This is likely related to the fact that PProDOP and similar polymers are known to be electron rich and easy to oxidize [12]. Preliminary tests of the lifetime of the devices indicated some loss in contrast during persistent switching (no open circuit pause) between 0 and −1.2 V, but most of the switching capability remained even after 1000 cycles (Supporting Information). We consider this quite normal for organic electrochromism and the lifetime can probably be improved by pulsed operation or by using other electrolytes [21].

## 4 Conclusions

In summary, we have shown how to fabricate nanostructures functionalized with an electrochromic polymer that improves the contrast of the intensity modulation in the blue spectral region. When switching blue structural colors on and off, we reach a maximum reflectivity change of at least 50%. The maximum change in transmittivity for the polymer on a planar electrode surface is around 75% at 521 nm and 60% in the blue. We note that there could potentially be polymers that improve the contrast in the green and red spectral regions as well, but we have focused on blue because it was until now clearly providing lower contrast. Besides the obvious relevance for reflective color display devices, the use of PProDOP can also improve the performance of other active devices operating in the blue spectral region. Further improvement may also be possible by more advanced synthesis protocols involving derivates of the monomer [22], copolymerization [19] etc.

## Supplementary Material

Supplementary Material Details
