# Supplementary material for: Enhanced electrochromic switching contrast in the blue by 3,4-propylenedioxypyrrole – implementation on structural colors
Source: Nanophotonics. 2023 Jan 12;12(8):1591–9. doi: 10.1515/nanoph-2022-0624 (PMC11501557; doi:10.1515/nanoph-2022-0624)
Supplement: Supplementary file 1 — Supplementary Material Details [file j_nanoph-2022-0624_suppl.pdf]

Enhanced Electrochromic Switching Contrast in the Blue by 3,4-propylenedioxypyrrole - Implementation on Structural Colors

Oliver Olsson, Marika Gugole and Andreas Dahlin.

Department of Chemistry and Chemical Engineering, Chalmers University of Technology, 41296 Gothenburg, Sweden.

We here give some details on factors influencing the preparation of the PProDOP films and speculate on what causes these differences. Note that the main text describes the recipe that worked best overall.

When using pure anhydrous PC the polymer was produced on the surface, but did not adhere well. Added water facilitated better adhesion of the polymer to the gold together with a higher polymerization rate. Thicker samples were obtained for the same polymerization time and a higher current was measured. This “water effect” have previously been explained for pyrrole in acetonitrile where it is suggested that the water acts as a proton scavenger during the polymerization. If a proton scavenger is not present to capture the protons produced during the polymerization the protons could react with the monomer and render a partly non-conjugated polymer that passivates the electrode. (See ref. 18 in main text.)

We also noted that the films should ideally be cleaned by vigorous shaking in isopropanol. If not shaken but carefully dipped in the isopropanol, the film became less uniform which could be due to unadhered polymer precipitating on the surface.

While electropolymerization with added water always resulted in the formation of an organic coating on the surfaces, the optical activity of the films was sometimes poor, i.e. their absorption did not increase as much when reducing the films to their pristine state. When polymerizing using PC (anhydrous 99.7%) with added water, the electrochromic performance of the finished films was poorer. We here refer to such films as “bad”. When using PC (ReagentPlus®) with added water the electrochromic performance of the films was better (and is referred to as “good”). However, the water concentration should not be too high as there seemed to be an optimum optical performance at slightly below 0.2% (Figure S1).

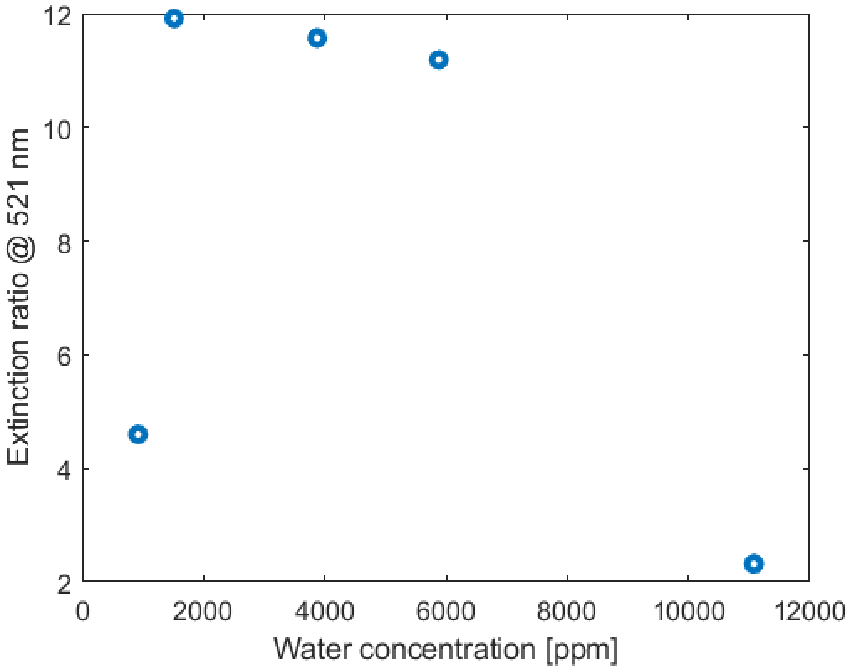

Figure S1 Measured extinction ratio (i.e. a measure of contrast) for PProDOP films polymerized in the presence of different amounts of water. The conditions were otherwise as described in the main text and the polymerization time was 300 s in all cases.

We also noted that the bad films had a low charge capacity per amount of deposited polymer of ~3% (the ratio of charge for a switch to charge accumulated during polymerization), while the good films had a higher value (~8%). The theoretical value is ~11% if every 4th monomer is accompanied with an ion (monovalent ion and two electrons per monomer).

The contrast was diminished (dark state became more transparent) with electrochemical switches when using an electrolyte not prepared with clean chemicals (anhydrous PC, battery grade $LiClO_4$) and stored in a glovebox. This could be attributed to water and/or oxygen impurities in the ambient-stored electrolyte. Therefore, deposition with cyclic voltammetry would have been a worse polymerization method since the film then switches during the polymerization and the electrolyte used in the polymerization does need to contain some water.

FTIR for the good samples displayed a sharp peak at 1565 cm^{-1} while the bad samples had the peak split (1576 cm^{-1} and 1548cm^{-1}). Example spectra are shown in Figure S2. One reason the bad films could not attain a strongly colored state could be because of deposition of partly non-

conjugated polymer (discussed above) facilitated by some additives in the anhydrous version of PC. Another reason could be the inclusion of the monomer in the polymer film. The monomer displays a similar peak at 1548 cm^{-1} which the bad films also exhibit but not the good films (Figure S2).

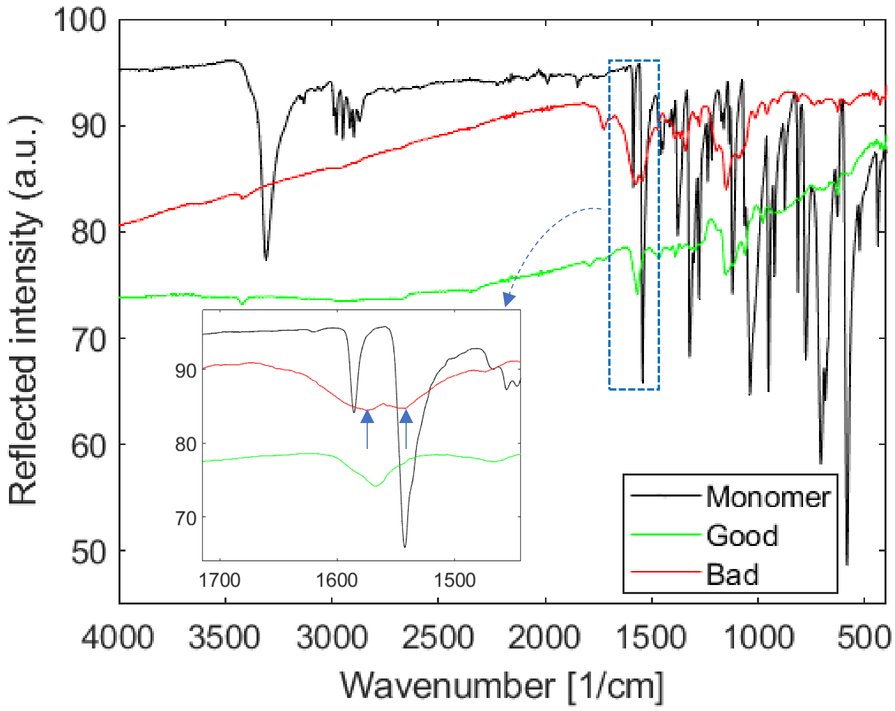

Figure S2 FTIR spectra of good and bad films, including also the ProDOP monomer. The zoomed in region shows how a peak for the good films splits into two for the bad films.

Note that even if these results give an idea of how to optimize the probability of getting good PProDOP films, further work is needed to obtain a fully reliable method when polymerizing on metals such as gold.

The lifetime of the devices is closely related to the film quality. Preliminary results of the contrast loss from a persistent switching are shown in Figure S3.

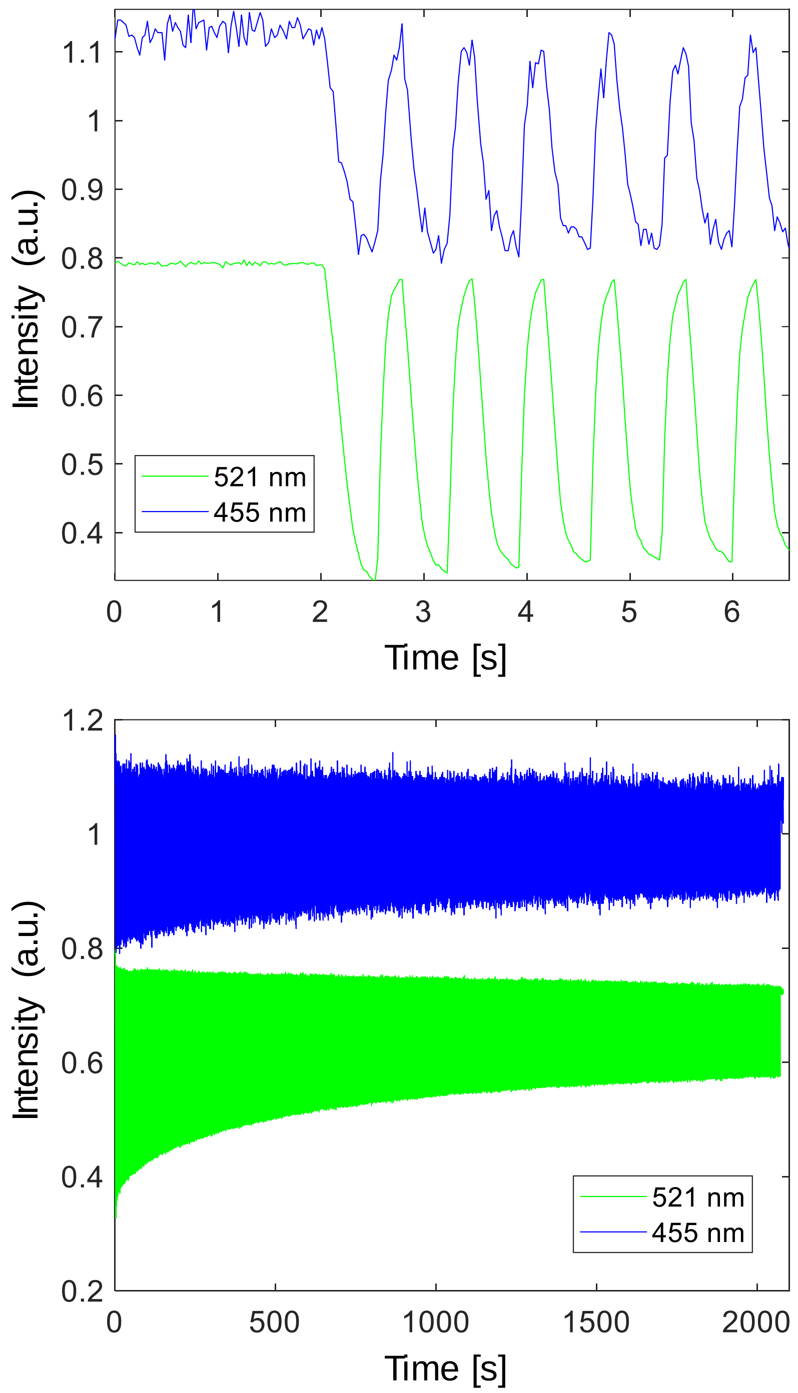

Figure S3 Lifetime test on a PProDOP film (300 s electropolymerization). The intensity is measured in transmission mode. The voltage is switched between −1.2 V and 0 V. Curves have been offset for clarity. The upper plot shows the first few switches, while the lower plot shows 1000 cycles.
